# Training the Gut for Athletes

**DOI:** 10.1007/s40279-017-0690-6

**Published:** 2017-03-22

**Authors:** Asker E. Jeukendrup

**Affiliations:** School of Sport, Exercise and Health Sciences, Loughborough University, Ashby Road, Loughborough, Leicestershire LE11 3TU UK

## Abstract

The gastrointestinal (GI) tract plays a critical role in delivering carbohydrate and fluid during prolonged exercise and can therefore be a major determinant of performance. The incidence of GI problems in athletes participating in endurance events is high, indicating that GI function is not always optimal in those conditions. A substantial body of evidence suggests that the GI system is highly adaptable. Gastric emptying as well as stomach comfort can be “trained” and perceptions of fullness decreased; some studies have suggested that nutrient-specific increases in gastric emptying may occur. Evidence also shows that diet has an impact on the capacity of the intestine to absorb nutrients. Again, the adaptations that occur appear to be nutrient specific. For example, a high-carbohydrate diet will increase the density of sodium-dependent glucose-1 (SGLT1) transporters in the intestine as well as the activity of the transporter, allowing greater carbohydrate absorption and oxidation during exercise. It is also likely that, when such adaptations occur, the chances of developing GI distress are smaller. Future studies should include more human studies and focus on a number of areas, including the most effective methods to induce gut adaptations and the timeline of adaptations. To develop effective strategies, a better understanding of the exact mechanisms underlying these adaptations is important. It is clear that “nutritional training” can improve gastric emptying and absorption and likely reduce the chances and/or severity of GI problems, thereby improving endurance performance as well as providing a better experience for the athlete. The gut is an important organ for endurance athletes and should be trained for the conditions in which it will be required to function.

## Training the Gut

Athletes often underestimate the importance of the gastrointestinal (GI) tract. The supply of exogenous fluid and carbohydrate sources can be critical to performance, especially during prolonged exercise [1]. In addition, GI symptoms such as bloating, cramping, diarrhea, and vomiting are common in many sports, especially endurance sports [2]. Without a well-functioning GI system, delivery of nutrients will be impaired and a range of GI symptoms may develop. Clearly, the intestinal tract is highly adaptable, and it has been suggested that targeted training of the intestinal tract may improve the delivery of nutrients during exercise while at the same time alleviating some (or all) of the symptoms [3]. This training, sometimes referred to as “training the gut,” has received relatively little attention in the literature, and to the best of my knowledge there are no dedicated review articles on this topic. I provide a more detailed overview of the evidence that the GI system can adapt through nutritional training.

## Gastric Emptying and “Stomach Training”

Gastric emptying is an important step towards delivering exogenous carbohydrate and fluids to the working muscle. Anecdotally, athletes complain about drinks accumulating in the stomach and feeling bloated, especially during high-intensity [4] or very prolonged exercise in hot conditions. Dehydration can contribute to this phenomenon and worsen complaints [4, 5]. Anecdotal evidence also shows that the stomach can adapt to ingesting large volumes of fluid, solids, or combinations. For example, serious contestants in eating competitions are known to “train” their stomach to hold larger volumes of food with less discomfort and—through regular training—are able to eat volumes of food within a small time window that are unthinkable for the average and untrained person. The current all-time record is 69 hot dogs (with bun) in 10 min. To achieve this, competitive eaters train using a variety of methods: chewing large pieces of chewing gum for longer periods of time or stomach extension by drinking fluids or by eating the competition foods. Volumes are progressively increased, and it takes many weeks to reach a level where these eaters can be competitive. This demonstrates the adaptability of the stomach. Conducting this “stomach training” has two main effects: (1) the stomach can extend and contain more food and (2) a full stomach is better tolerated and is not perceived as so full. Both aspects could be relevant to an exercise situation.

Current guidelines recommend fluid intakes during exercise that prevent 2% dehydration (2% of body weight). Recommended fluid intake can be substantial, especially in trained athletes and hot conditions when sweat rates are high. Such high intakes can cause discomfort and in some cases GI problems. Therefore, athletes are generally simultaneously managing GI comfort, hydration, and carbohydrate delivery. I and others have recommended training for these higher intakes to reduce discomfort and the chance of GI distress [3, 6, 7]. However, very few studies have directly investigated such effects of “nutritional training of the stomach.”

Lambert et al. [8] showed that trained runners were able to comfortably tolerate ingestion of a carbohydrate–electrolyte solution at a rate approximately equal to their sweat rate during 90 min of running at 65% maximum oxygen uptake (*V*O_2max_) in a ~25 °C, 30% relative humidity (RH) environment. Interestingly, they observed that stomach comfort significantly improved over time by practicing these high intakes. It must be noted that this improved comfort occurred without measurable changes in the rate of gastric emptying [8]. Perhaps the stomach adapted by extending the stomach walls to allow greater space for fluid. This would likely reduce feelings of stomach discomfort and reduce the stimulus for faster gastric emptying. Training for intake of larger volumes could be an effective strategy to avoid these problems in races, particularly for athletes who experience GI discomfort even when ingesting relatively small volumes.

Studies have also demonstrated that gastric emptying of carbohydrate can be accelerated by increasing dietary intake of that carbohydrate. Cunningham et al. [9] supplemented the diet of two groups of volunteers with glucose 400 g per day for 3 days. The half emptying time (*t*_½_) for the glucose test meal was significantly faster after the standard diet had been supplemented with glucose compared with the standard diet alone (median 20.7 min [range 4.6–36.8] vs. 29.1 [range 19.8–38.4]). Interestingly, the gastric emptying of a protein drink was unchanged (median 18.0 min [range 12.5–23.6] vs. 16.1 [range 9.6–22.7]). The authors concluded that rapid and specific adaptation of the small intestinal regulatory mechanisms for gastric emptying of nutrient solutions can occur in response to increases in dietary load. Another study showed that supplementing a standard diet with glucose 440 g per day for 4–7 days accelerated gastric emptying of both glucose and fructose (*t*_½_ 82 ± 8 vs. 106 ± 10 min for glucose and 73 ± 9 vs. 106 ± 9 min for fructose) [10]. Plasma glucose-dependent insulinotropic peptide (GIP) concentrations were higher during the glucose-supplemented diet; thus the authors concluded that the gastric emptying of both glucose and fructose was accelerated probably as a result of reduced feedback inhibition from intestinal luminal receptors [10].

One study showed that daily ingestion of fructose 120 g for 3 days accelerated gastric emptying of fructose but not of glucose [11]. It appears that the relatively short duration of the dietary manipulation (3 days) was sufficient to cause adaptations in gastric emptying.

Such observations are not specific for carbohydrate. Studies have demonstrated that a higher-fat diet stimulated gastric emptying. Cunningham et al. [12] demonstrated that gastric emptying of a test meal was accelerated after 7 days of a higher-fat diet (258 g/day). Reductions in *t*_½_ of a test meal in response to the intervention reached significance after 14 days. Similar trends were observed after 4 days but did not reach statistical significance, suggesting that adaptations to fat in the diet may be slower than responses to carbohydrate. Castiglione et al. [13] demonstrated a similar adaptation after 14 days of a high-fat diet and reported that these effects were highly specific to fats and that a carbohydrate meal was emptied at the same rate before and after a high-fat diet.

Adaptations are likely explained by desensitization of nutrient receptors and reduced feedback inhibition of gastric emptying. However, it is also possible that increased absorption results in reduced exposure of receptors to nutrients. Sections 6 and 7 provide evidence of increased absorption of nutrients in response to changes in diet.

### Stomach Training: Summary

Some studies have clearly demonstrated that specific nutritional challenges result in specific adaptations of gastric emptying to that challenge. For example, increased dietary glucose intake increases the gastric emptying of glucose but not protein, and increased fat intake results in faster gastric emptying of fats but not carbohydrate. Very few studies have specifically trained the gut to improve tolerance and gastric emptying during exercise, but the results generally look promising. Effects have been observed after 3 days of dietary manipulation.

## Intestinal Sugar Transport

**Fig. 1:**
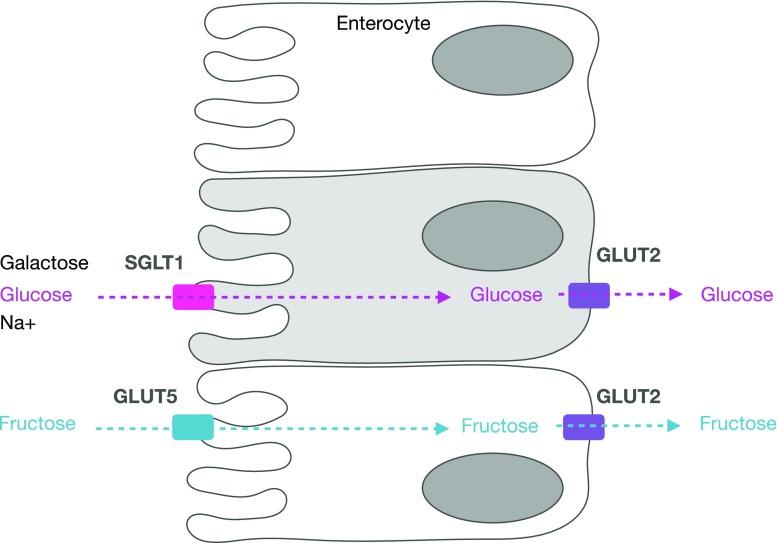
Absorption of glucose and fructose. Glucose and fructose are absorbed from the intestinal lumen (on the left) through the enterocyte (luminal and basolateral membrane) into the circulation (on the right), via different pathways involving SGLT1 and GLUT5, respectively. *SGLT1* sodium-dependent glucose transporter 1, *GLUT5* glucose transporter 5 (fructose transporter), *GLUT2* glucose transporter 2

Once emptied from the stomach, most fluid and sugar absorption will take place in the duodenum and jejunum. Glucose and galactose are transported across the luminal membrane of enterocytes by the sodium-dependent glucose transporter (SGLT)-1 (Fig. 1).

### Sodium-Dependent Glucose Transporter (SGLT)-1

Absorption of glucose (and galactose) is coupled with sodium transport and the associated electrochemical gradient. An Na/K^+^ ATP-ase, located at the basolateral membrane, is responsible for maintaining the electrochemical gradient. There have been suggestions that glucose transporter (GLUT)-2, another transporter, can be recruited to the luminal membrane when high concentrations of glucose are present in the lumen [14, 15]. This theory of additional facilitated glucose transport remains controversial, and SGLT1 is generally believed to be responsible for the vast majority of absorption of dietary sugars [16].

In most mammalian studies, SGLT1 has been shown to be expressed on the brush border of enterocytes [17–21]. Expression levels are usually highest in the jejunum, followed by the duodenum and ileum [22]. SGLT1 is not expressed in the large intestine [22].

### Glucose Transporter (GLUT)-5

Fructose uses a different transporter (GLUT5) to glucose that is not sodium dependent and is highly specific to fructose. The regulation of GLUT5 is more rapid than the regulation of SGLT1. Changes in fructose transport are typically paralleled by similar changes in GLUT5 messenger RNA (mRNA) and protein abundance. In rats, GLUT5 mRNA doubles within 3 h after intestinal perfusion with a fructose solution [23]. It must be noted that these effects have only been demonstrated at unnaturally high fructose intakes (at least 30% of energy in the diet coming from fructose, whereas a typical intake in a Western diet is around 9%).

### GLUT2

From the enterocyte to the systemic circulation, the sugars need to pass the basolateral membrane. All three monosaccharides use the bidirectional transporter GLUT2, which is also sodium independent. The capacity of GLUT2 to transport glucose across a concentration gradient is believed to be very large [14, 15].

### Other Transporters

There is little evidence for other carbohydrate transporters in addition to SGLT1 and GLUT5 transporters at the luminal membrane and GLUT2 at the basolateral membrane. There have been suggestions of other transporters, but it seems that if they exist they will be relatively unimportant for transport of carbohydrates from a quantitative point of view. Since GLUT2 does not seem to be limiting, I focus primarily on SGLT1 and GLUT5.

## Carbohydrate Transporters and Glucose Transport During Exercise

Regulation of carbohydrate transport proteins is essential for the provision of glucose to the body in resting conditions. Furthermore, during exercise, when exogenous delivery of carbohydrate may be important for performance, the transporters will be responsible for glucose delivery to the working muscle. Exercise studies have provided indirect but strong evidence that the delivery of carbohydrate is limited by the transport capacity of SGLT1 (for reviews, see Jeukendrup [1, 6, 7] and Jeukendrup and McLaughlin [3]). A recent review based primarily on more direct measurements in animals also concluded that the intestine has the capacity to absorb glucose via basal levels of SGLT1 but that this capacity becomes limiting when dietary carbohydrate exceeds a certain level [24].

**Fig. 2:**
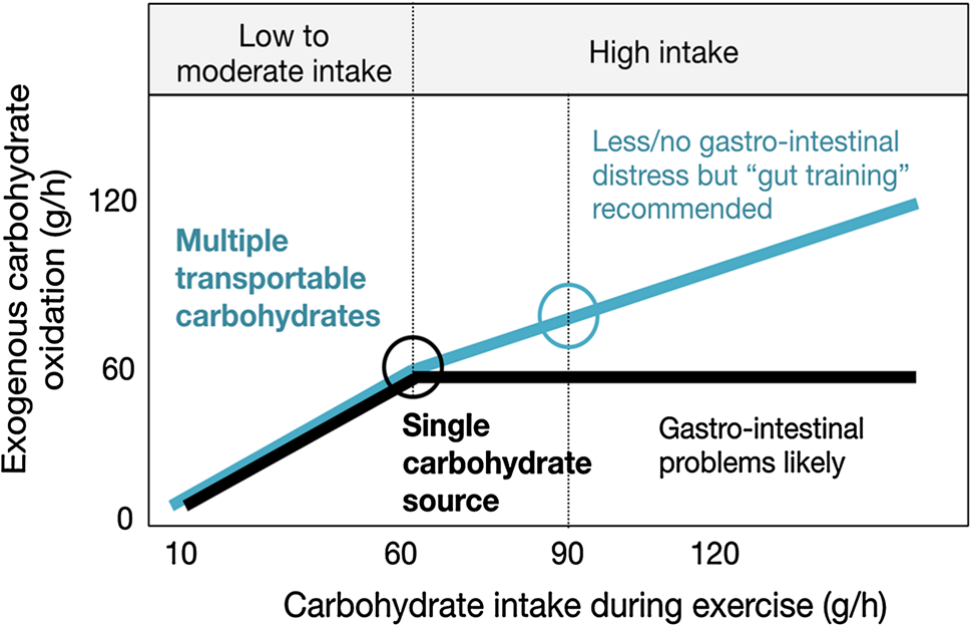
Schematic of exogenous carbohydrate oxidation from a single carbohydrate (*black*) and multiple transportable carbohydrates (*blue*), based on data presented elsewhere [3, 7, 51, 52]. It is clear that higher oxidation rates can be achieved with multiple transportable carbohydrates, especially at high intakes. At intakes up to 60 g/h, there is no difference between single and multiple transportable carbohydrates, but when intake increases above 60 g/h and the sodium-dependent glucose transporter 1 (SGLT1) becomes saturated, added fructose will result in higher exogenous carbohydrate oxidation rates. The recommended intake for single and multiple transportable carbohydrates are indicated with a *circle*. If single carbohydrate sources are ingested at rates higher than 60 g/h, gastrointestinal problems are likely. With multiple transportable carbohydrates, fewer symptoms have been observed, but “training the gut” (and getting used to high intakes) is recommended

At ingestion rates over 60–70 g of carbohydrate per h (glucose, sucrose, maltose, maltodextrin, starch), exogenous carbohydrate oxidation peaks around 60 g/h (Fig. 2) [1, 3, 6, 7]. Even ingestion at 144 g/h [25] or 180 g/h [26] did not increase exogenous carbohydrate oxidation rates much above 60 g/h. Because this limitation was not caused by gastric emptying, muscle glucose uptake, or liver glycogen storage, it was deduced that absorption had to be limiting [27]. When fructose was ingested in addition to larger amounts of glucose, carbohydrate oxidation rates were elevated above 60 g/h [28]. These studies strongly suggested that glucose transport across the epithelial cell was the limiting factor and that the maximal transport capacity of SGLT1 was reached [29]. Because there also appears to be a dose–response relationship between carbohydrate intake and performance [30–32], and it is likely that a reduced capacity of the intestine in combination with a higher carbohydrate intake may result in GI distress [2], the search for ways to increase the capacity to absorb carbohydrate continues.

Training the gut has been proposed as a way to increase SGLT1 transporter number and/or activity, but evidence in humans thus far is limited [6].

Using a segmental perfusion technique, Shi et al. [33] reported a close relationship between water absorption and solute absorption in the duodenojejunum, especially when multiple transportable substrates are present (i.e., glucose, sucrose, glycine, Na^+^). We confirmed this in humans during exercise: multiple transportable carbohydrates increased carbohydrate absorption and oxidation and this was associated with increased fluid absorption [34]. Therefore, one other benefit of increasing the transport capacity for carbohydrate is that fluid intake is likely to also be improved (for a given carbohydrate intake). Improved fluid absorption can help prevent dehydration (and dehydration-induced reductions in performance), but more complete absorption may also reduce the chances of GI discomfort [2].

To develop practical recommendations, it is important to understand the regulation of intestinal glucose transport. I therefore discuss the regulation in more detail before providing suggestions for practical implication.

## Regulation of Intestinal Glucose Transport

Regulation of glucose absorption has been shown to be directly linked to the expression of SGLT1 protein. Bob Crane proposed the existence of an Na^+^/glucose co-transport in 1960 at the Symposium on Membrane Transport and Metabolism in Prague [35], but the actual transporter was not identified until the 1980s [36]. Studies in the 1960s also observed that dietary carbohydrate intake can influence the capacity to absorb glucose [37]. In 1983, it was demonstrated that intestinal transporters were upregulated and downregulated depending on dietary composition [38]. At least in rats, it appears that dietary changes do not have to be extreme to observe effects on absorption, and these effects have been seen not only for sugars but also for amino acids [38]. Increases in absorption have been observed in as little as 0.5 days in rats [38]. It was also observed very early on that digestive enzymes were upregulated in response to dietary composition. For example, Deren et al. [39] demonstrated in 1967 that rats who were fasted for 3 days displayed fourfold increases in sucrase and maltase activity in response to a sucrose diet compared with a casein diet [39]. This was correlated with increases in sucrose hydrolysis and in fructose absorption.

When sugar transporters were identified in the gut in the 1980s, studies started to measure changes in SGLT1 content and activity in response to diet. A number of rodent models [18, 40] have shown that both the activity and the abundance of SGLT1 is regulated by dietary carbohydrate intake. It is clear that SGLT1 protein responds to glucose concentrations in the lumen. However, SGLT1 was stimulated to the same degree when membrane-impermeable glucose analogues were used [41], suggesting that a glucose sensor detects glucose or its analogues, initiating the upregulation of the SGLT1 transporters.

### Sensing Mechanism

Specialized cells (L cells and K cells) in the intestinal luminal membrane have been shown to express taste receptor cells. In particular, it has been demonstrated that T1R2 and T1R3 receptors detect sweetness. The T1R2 and T1R3 cells are coupled through a G-protein (alpha-gustducin) to a cascade of downstream cellular events that ultimately lead to upregulation of SGLT1. A more detailed discussion of the potential pathways involved is provided in the following sections.

### Sweeteners and Other Analogues

SGLT1 is upregulated in response not only to dietary carbohydrate but also to sweeteners. Margolskee et al. [17] confirmed earlier findings that SGLT1 protein expression in wild-type mice receiving a diet supplemented with carbohydrate almost doubled compared with mice receiving a low-carbohydrate diet. However, SGLT1 expression also doubled when the low-carbohydrate diet was supplemented with the sweeteners sucralose, acesulfame K, or saccharine, but not when supplemented with aspartame. The observation that aspartame had no effect is not surprising because it is known that mice do not experience aspartame as sweet.

### Other Dietary Constituents that Regulate Intestinal Glucose Transport

A number of dietary constituents have been implicated in the regulation of glucose transport. Sodium chloride consumption appears to modulate intestinal glucose transport. Studies suggest that chronically elevated luminal concentrations of glucose and sodium will lead to increased expression of the SGLT1 protein [42]. There are still many questions about the mechanisms and whether the effects of sodium and glucose are additive [43].

Dietary fiber is another constituent with potential effects, but studies have been inconclusive: some studies show a decrease, some show no change, and some show an increase in intestinal glucose transport with increasing dietary fiber intake [43]. Fiber is a broad term used to describe vastly different characteristics, and fiber can have effects on gastric emptying, motility, and the composition and structure of the intestinal tract. Therefore, it may not be surprising that results of studies have been inconclusive.

To the best of my knowledge, no studies in humans have investigated the effects of dietary constituents on intestinal glucose absorption. Therefore, developing firm guidelines in the absence of these findings would be premature.

### Molecular Mechanisms

SGLT1 protein is upregulated in response to a number of stimuli, including but not limited to glucose and galactose: 3-*O*-methylglucose (non-metabolizable substrate of SGLT1) and fructose (not a substrate of SGLT1). Upregulation of the SGLT1 protein depends on the availability of these sugars, but metabolism of these sugars is not necessary. The fact that SGLT1 expression responds to glucose analogues and sugars not transported by SGLT1 suggests there is a separate receptor that detects these glucose analogues.

**Fig. 3:**
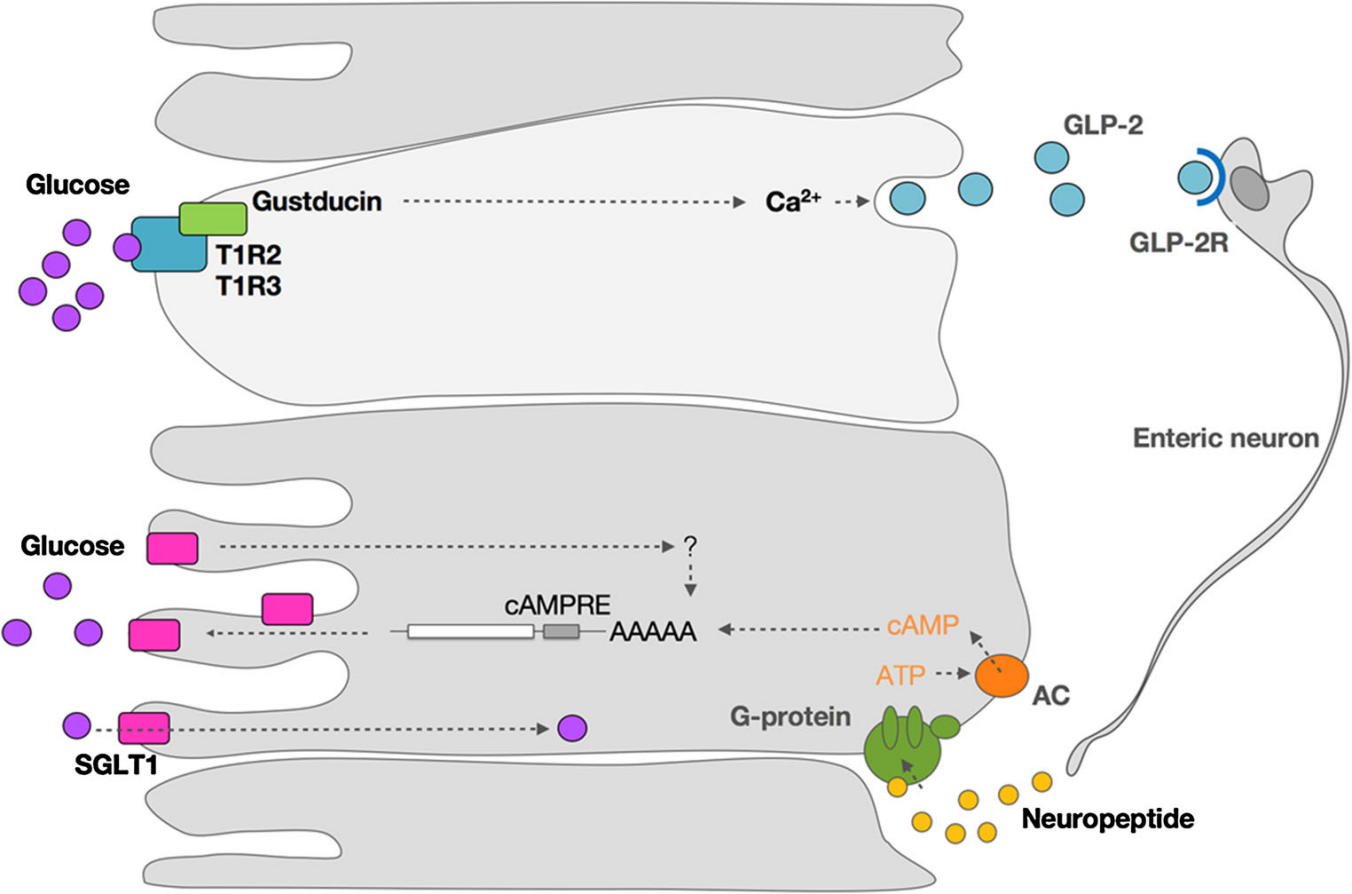
A proposed mechanism for upregulation of sodium-dependent glucose transporter 1 (SGLT1) protein. Sweet receptors T1R2 + T1R3, expressed on the luminal membrane of villus endocrine cells, sense luminal concentration of glucose. When this glucose concentration reaches a threshold, it activates a signaling cascade in endocrine cells that involves T1R2 + T1R3 receptors, gustducin, and other signaling elements. This will result in the secretion of GLP-1, GLP-2, and GIP. GLP-2 binding to its receptor GLP-2R on enteric neurons elicits an action potential. This stimulus, in turn, is transmitted to sub-epithelial regions by axonal projections, which will evoke the release of a neuropeptide in the absorptive enterocytes. The binding of this neuropeptide to its receptor increases intracellular cAMP concentrations, thereby increasing the stability of mRNA of SGLT1 and increasing the SGLT1 protein concentration. *AAAAA* amino acid chain, *AC* adenylate cyclase, *cAMP* cycling AMP, *cAMPRE* cyclic AMP response element, *GIP* glucose-dependent insulinotropic peptide, *GLP* glucagon-like peptide, *GLP-2R* receptor for GLP-2, *mRNA* messenger RNA., *SGLT1* sodium dependent glucose transporter 1, *T1R2* *+* *T1R3* taste receptor formed as a dimer of the T1R2 and T1R3 proteins. Adapted from Shirazi-Beechey et al. [24] with permission

Studies have suggested that the sugar-mediated upregulation of SGLT1 is likely to involve a G-protein-coupled second messenger pathway [41, 44] (Fig. 3). More recently, it was demonstrated in mice that T1R3 and gustducin are expressed in enteroendocrine cells and are required for the expression of SGLT1 in vivo in response to luminal sugars or sweeteners [17]. SGLT1, on the other hand, is expressed in enterocytes (Fig. 3). This means that a signaling event must take place between chemosensory enteroendocrine cells and absorptive enterocytes. It is known that enteroendocrine cells can secrete endocrine hormones such as cholecystokinin (CCK), peptide tyrosine tyrosine (PYY), neurotensin, glucagon-like peptide (GLP)-1, GLP-2, and GIP. The incretins GLP-1 and GIP are secreted in response to dietary sugars and influence glucose transport, metabolism, and homeostasis. Shirazi-Beechey et al. [24] described a possible pathway of regulation. Sweet receptors T1R2 + T1R3, expressed on the luminal membrane of villus endocrine cells, sense the luminal concentration of glucose. When this glucose concentration reaches a threshold, it activates a signaling cascade in endocrine cells that involves T1R2 + T1R3 receptors, gustducin and other signaling elements. This will result in the secretion of GLP-1, GLP-2, and GIP. The binding of GLP-2 to its receptor on enteric neurons elicits an action potential. This stimulus is transmitted to sub-epithelial regions by axonal projections. This will evoke the release of a neuropeptide in the absorptive enterocytes. The binding of this neuropeptide to its receptor results in increased intracellular cyclic adenosine monophosphate (cAMP) concentrations, thereby increasing the stability of mRNA of SGLT1 and increasing the SGLT1 protein concentration.

GIP has been shown to directly regulate SGLT1 and enhance absorption in the mouse jejunum. In turn, increased glucose absorption also has an effect on GIP secretion [45]. Recent studies have demonstrated that a GIP receptor knockout had marked effects on SGLT1 expression, suggesting that GIP plays an important role in the upregulation of SGLT1 [46].

Several aspects of this mechanism remain untested, and some aspects have been disputed [16], but it is clear that somehow luminal glucose is sensed and—through a signaling cascade—SGLT1 function protein levels increase.

### Time Course

In mice, intestinal SGLT1 protein in brush–border membrane vesicles in the mid small intestine increased 1.9-fold after 2 weeks of a high-carbohydrate diet [17]. In a study of horses, which are believed to be slow adapters to an increase in carbohydrate, SGLT1 protein expression from intestinal biopsies was increased after just 1 week of high-carbohydrate feeding, and the abundance increased further after 1 and 2 months on the diet. Piglets who received a higher-carbohydrate diet for 3 days showed increases of SGLT1 protein as well as glucose absorption [47].

Although no direct human studies exist, a large number of animal studies suggest that the time course of changes in SGLT1 expression is relatively rapid. Several studies have observed significant changes after only a few days of dietary change [17]. It seems therefore reasonable to suggest that several days of a high-carbohydrate intake can increase SGLT1 content and the capacity to absorb glucose, but more prolonged exposure to the diet could result in greater adaptations.

## Regulation of Absorption in Athletes

An elegant study by Cox et al. [48] gives us the most important clues today that diet manipulation can result in improved delivery of carbohydrate during exercise. In this study, 16 endurance-trained cyclists were divided into a high-carbohydrate and a control group. For 28 days, both groups trained (16 h/week) and their performance improved as a result of this training. Both groups received a diet with a moderate carbohydrate content (5 g/kg/day). The high-carbohydrate group were supplemented with an additional 1.5 g/kg per hour of exercise performed daily. The carbohydrate supplement was provided mainly in the form of a glucose drink. In addition, they received carbohydrate-rich foods to meet the hourly demands of exercise. The control group also received a nutritional supplement, but this was composed of fat- and protein-rich foods with limited carbohydrate content. Subjects in the high-carbohydrate groups consumed the supplements before, during, and immediately after exercise. The cyclists in the control group consumed their supplement after exercise. On average, the carbohydrate-supplemented group had a high daily carbohydrate intake of 8.5 g/kg, whereas the control groups consumed 5.3 g/kg/day.

Before and after the 28-day training period, all subjects performed an exercise trial in which they received a 10% carbohydrate solution. Isotopic tracers were used to measure the oxidation of the exogenous carbohydrate. It was observed that exogenous carbohydrate oxidation was improved after the carbohydrate-supplemented diet. The most likely explanation is an increase in the ability to absorb carbohydrate as a result of an upregulation of SGLT1 transporters. It was concluded that, for athletes who compete in endurance events, where exogenous carbohydrate is an important energy source and there is ample opportunity to ingest carbohydrate, this higher carbohydrate intake approach may be beneficial [6, 7, 48].

It has become clear that an increase in dietary carbohydrate intake can increase the abundance and activity of intestinal SGLT1 transporters and that this results in an improved capacity to absorb carbohydrate. The reverse may also be true. With carbohydrate restriction through reducing carbohydrate intake, high-fat, or even ketogenic diets, or by reducing total energy intake, the daily carbohydrate intake can become very low. Studies in lambs have demonstrated that, as the diet changes from milk to grass, so the rumen, where dietary carbohydrates are fermented into volatile fatty acids, develops. Rumen formation effectively prevents the delivery of monosaccharides to the intestine. As a result, there is a marked decrease in both the SGLT1 protein content of the intestine as well as the capacity of the small intestine to absorb carbohydrate [49, 50].

## Gastrointestinal Problems

GI problems are very common amongst athletes, and 30–50% of all athletes experience such problems regularly [2]. The causes are still largely unknown but appear to be partly genetically determined and highly individual [2]. The mechanisms are likely to be different for upper and lower GI problems. The symptoms are more likely to occur and are exacerbated by hot weather conditions and dehydration [2].

Although a link with nutrition intake is not always found, certain practices have been found to correlate with the incidence of GI problems: fiber intake, fat intake, and highly concentrated carbohydrate solutions seem to increase the prevalence of GI problems.

There are probably several reasons for these problems, but two important and common reasons may be a bloated feeling and reduced gastric emptying during prolonged exercise, and diarrhea as a result of osmotic shifts.

It is thought that training the gut may alleviate some of these symptoms, perhaps by improving gastric emptying and the perception of fullness (reduced bloating), improving tolerance of larger volumes, and increasing the speed of absorption, causing less residual volume and smaller osmotic shifts [2].

## Practical Implications and Conclusions

**Fig. 4:**
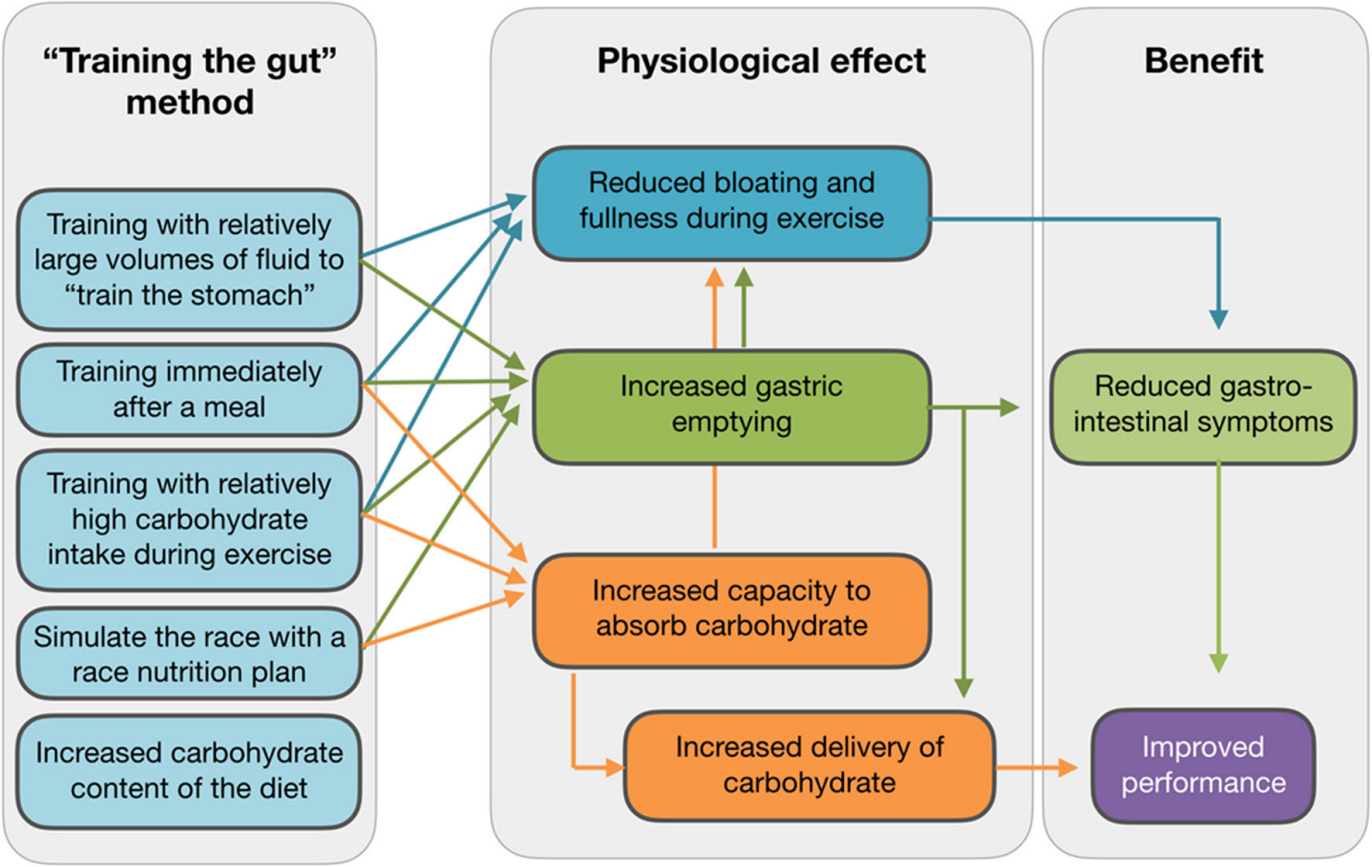
A summary of methods to “train the gut”, the adaptations that may occur in the gut, and implications for performance

A summary of practical implications is depicted in Fig. 4. While some extrapolations from animal studies are required, it is likely that adaptations in the human intestine are as rapid as those seen in other mammals. This means that several days and certainly 2 weeks of a high-carbohydrate diet would result in significant increases in the SGLT1 content of the intestinal lumen. Based on animal data, an increase in dietary carbohydrate from 40 to 70% could result in a doubling of SGLT1 transporters over a period of 2 weeks.

In addition to an increased absorptive capacity, it is essential that higher carbohydrate intakes can be tolerated and are also emptied from the stomach. Although it is generally believed that gastric emptying is not a limiting factor, it is likely that a combination of factors (for example, heat, high carbohydrate intake, and high-intensity exercise, which are all factors known to inhibit gastric emptying) will act together, thereby compromising gastric emptying. Therefore, it is important to practice a race nutritional strategy in training and get used to higher volumes of intake or higher carbohydrate intakes.

Most athletes consume a moderate- to high-carbohydrate diet, and it could therefore be argued that the benefits of increasing carbohydrate intake even more may only be small. At present, the link between daily carbohydrate intake and the transport capacity for glucose in the human intestine is uncertain. Perhaps the fact that we have seen little variation in the maximum exogenous carbohydrate oxidation rates in many years of research involving hundreds of participants is a sign that diet has relatively small effects on the maximal carbohydrate transport capacity of the gut. The fact that transport capacity has hardly ever exceeded 60 g/h, even in individuals, may be a sign that improvements may not be dramatic. On the other hand, research such as the study by Cox et al. [48] suggests that these transporters can be upregulated in a relatively short period of time.

Although the exact magnitude of effects in athletes who are already consuming a high-carbohydrate diet may be uncertain, it seems fair to conclude that those athletes who are not practicing a high-carbohydrate diet can benefit substantially. When athletes are carbohydrate restricting; following a low-carbohydrate, high-fat, or ketogenic diet; or are reducing energy intake to lose weight, the reduced daily carbohydrate load will likely reduce the capacity to absorb carbohydrates during competition. This could be a reason why these athletes anecdotally seem to report more GI problems. These athletes would be advised to include some high-carbohydrate days in their training.

Current guidelines recommend a carbohydrate intake up to about 60 g for exercise lasting for up to 2 h. When the exercise lasts ≥2 h, slightly greater amounts of carbohydrate (90 g/h) would be recommended, and these carbohydrates should consist of a mix of multiple transportable carbohydrates, e.g., glucose:fructose or maltodextrin:fructose. To obtain a carbohydrate intake of 90 g/h, athletes could “mix and match” to fulfil their personal preferences and take into account their tolerance [6, 7]. Since the gut is so adaptable, it seems wise to include training with high-carbohydrate intake into the weekly routine and regularly ingest carbohydrate during exercise. With these strategies, the gut may be trained to absorb and oxidize more carbohydrate, which in turn should result in less GI distress and better performance.
